# (Phospho)proteomic Profiling of Microsatellite Unstable CRC Cells Reveals Alterations in Nuclear Signaling and Cholesterol Metabolism Caused by Frameshift Mutation of NMD Regulator UPF3A

**DOI:** 10.3390/ijms21155234

**Published:** 2020-07-23

**Authors:** Malwina Michalak, Eva-Maria Katzenmaier, Nina Roeckel, Stefan M. Woerner, Vera Fuchs, Uwe Warnken, Yan P. Yuan, Peer Bork, Gabriele Neu-Yilik, Andreas Kulozik, Magnus von Knebel Doeberitz, Matthias Kloor, Jürgen Kopitz, Johannes Gebert

**Affiliations:** 1 Department of Applied Tumor Biology, Institute of Pathology, Heidelberg University Hospital, Im Neuenheimer Feld 224, 69120 Heidelberg, Germany; malwina.michalak@med.uni-heidelberg.de (M.M.); Eva-Maria.Katzenmaier@med.uni-heidelberg.de (E.-M.K.); Nina.blessing@roche.com (N.R.); Vera.Fuchs@med.uni-heidelberg.de (V.F.); magnus.knebel-doeberitz@med.uni-heidelberg.de (M.v.K.D.); Matthias.Kloor@med.uni-heidelberg.de (M.K.); juergen.kopitz@med.uni-heidelberg.de (J.K.); 2 Molecular Medicine Partnership Unit, Medical Faculty of the University of Heidelberg and European Molecular Biology Laboratory, 69120 Heidelberg, Germany; stefan.woerner@uni-heidelberg.de (S.M.W.); Peer.Bork@embl.org (P.B.); Gabriele.Neu-Yilik@med.uni-heidelberg.de (G.N.-Y.); Andreas.Kulozik@med.uni-heidelberg.de (A.K.); 3 Department of Pediatric Oncology, Hematology and Immunology, Children’s Hospital, University of Heidelberg, Im Neuenheimer Feld 430, 69120 Heidelberg, Germany; 4 Department of Internal Medicine I, Endocrinology and Metabolism, University of Heidelberg, Im Neuenheimer Feld 410, 69120 Heidelberg, Germany; 5 Clinical Cooperation Unit Neurooncology, DKFZ (German Cancer Research Center), Im Neuenheimer Feld 280, 69120 Heidelberg, Germany; u.warnken@dkfz-heidelberg.de; 6 Structural and Computational Biology Unit, European Molecular Biology Laboratory, Meyerhofstraße 1, 69117 Heidelberg, Germany; yuan@embl.de; 7 Max-Delbrück-Centre for Molecular Medicine, Robert-Rössle-Straße 10, 13125 Berlin, Germany; 8 Clinical Cooperation Unit Applied Tumor Biology, DKFZ (German Cancer Research Center) Heidelberg, Im Neuenheimer Feld 280, 69120 Heidelberg, Germany

**Keywords:** nonsense-mediated RNA decay, coding mononucleotide repeats, DNA mismatch repair, MSI tumorigenesis, UPF3A

## Abstract

DNA mismatch repair-deficient colorectal cancers (CRCs) accumulate numerous frameshift mutations at repetitive sequences recognized as microsatellite instability (MSI). When coding mononucleotide repeats (cMNRs) are affected, tumors accumulate frameshift mutations and premature termination codons (PTC) potentially leading to truncated proteins. Nonsense-mediated RNA decay (NMD) can degrade PTC-containing transcripts and protect from such faulty proteins. As it also regulates normal transcripts and cellular physiology, we tested whether NMD genes themselves are targets of MSI frameshift mutations. A high frequency of cMNR frameshift mutations in the *UPF3A* gene was found in MSI CRC cell lines (67.7%), MSI colorectal adenomas (55%) and carcinomas (63%). In normal colonic crypts, UPF3A expression was restricted to single chromogranin A-positive cells. SILAC-based proteomic analysis of KM12 CRC cells revealed UPF3A-dependent down-regulation of several enzymes involved in cholesterol biosynthesis. Furthermore, reconstituted UPF3A expression caused alterations of 85 phosphosites in 52 phosphoproteins. Most of them (38/52, 73%) reside in nuclear phosphoproteins involved in regulation of gene expression and RNA splicing. Since UPF3A mutations can modulate the (phospho)proteomic signature and expression of enzymes involved in cholesterol metabolism in CRC cells, UPF3A may influence other processes than NMD and loss of UPF3A expression might provide a growth advantage to MSI CRC cells.

## 1. Introduction

Microsatellite instability (MSI) is a genetic phenotype characterized by the accumulation of insertion/deletion mutations in short repetitive sequences. This is observed in the majority of tumors associated with hereditary nonpolyposis colorectal cancer (HNPCC, Lynch syndrome) but also occurs in approximately 15% of sporadic colorectal carcinomas. At the molecular level, MSI is caused by functional inactivation of the cellular DNA mismatch repair (MMR) system. In HNPCC-associated tumors, MMR deficiency arises due to germline and somatic mutations in one of several DNA MMR-genes (*MLH1, MSH2, MSH6,* and *PMS2*) whereas epigenetic silencing of the *MLH1* gene accounts for the development of sporadic MSI tumors. In contrast to their microsatellite stable counterparts, colorectal cancers with high level of microsatellite instability (MSI) show distinct clinico-histopathological features including predominant proximal localization, strong lymphocytic infiltration, better prognosis and altered chemoresponsiveness [1,2,3]. 

Frameshift mutations in coding region microsatellites (coding mononucleotide repeats, cMNRs) of specific genes are thought to contribute to these clinico-histopathological features. Accordingly, many studies have been performed to predict, identify, and validate target genes potentially involved in MSI tumorigenesis [4,5,6]. Examples include cMNR-harboring genes encoding proteins of major cellular pathways such as signal transduction (TGFBR2, ACVR2), apoptosis (BAX), and transcription (TCF4). At the transcriptional level these cMNR insertion/deletion mutations lead to shifts in the translational reading frames. These alternative reading frames often contain premature translation termination codons (PTCs) and potentially induce the synthesis of truncated and/or functionally altered proteins. Several cellular control mechanisms suppress the expression of such transcripts, the best characterized of these being the nonsense-mediated mRNA decay pathway (NMD). NMD recognizes mRNAs with PTCs located more than 50–55 base pairs upstream of the last exon-exon junction and initiates their degradation. This task is accomplished by several proteins namely the Upf (Up-frameshift) proteins UPF1, UPF2, UPF3A, and UPF3B, the SMG (Suppressor with Morphological effect on Genitalia) factors SMG1, SMG5, SMG6, SMG7, SMG8, and SMG9 as well as structural and peripheral components of the exon junction complex (Y14, MAGOH, RNPS1, eIF4AIII, CASC3, P29) [7,8]. The phosphoinositide 3-kinase (PI3K) such as SMG1 forms together with SMG8 and SMG9 a kinase complex that specifically regulates phosphorylation of UPF1 at several N-and C-terminal S/T-Q-sites. SMG5, SMG6, and SMG7 have been reported to be involved in dephosphorylation of UPF1. However, they also initiate decay of NMD target mRNAs. SMG6 is an endonuclease that cleaves PTC-containing mRNAs near the PTC. After translation termination at PTC the SMG5/7 complex recruits after translation termination at a PTC the cytoplasmic 3′-poly(A)-tail deadenylase CCR4/NOT complex which in turn initiates decapping and enables both 5′ and 3′ exonucleolytic decay of the RNA body [9].

Among these NMD pathway components UPF3 stands out because it exists in two paralogs, UPF3A and UPF3B that differ in their ability to regulate NMD and translational efficiency [10]. UPF3B is a bona fide NMD activator that has been reported to regulate translation termination and modulate ribosome recycling [11]. In contrast, its sister paralog UPF3A is a powerful NMD repressor thereby acting as a regulator of gene expression [12].

In addition to acting as a quality control system that rids cells of aberrant mRNAs with crippled protein coding potential, numerous studies revealed that NMD constitutes an important post-transcriptional layer of gene expression control involved in the regulation of many different biological pathways [13]. In particular, NMD targets at least 10% of normal mammalian mRNAs to modulate appropriate cellular responses—adaptation, differentiation, or death—to environmental changes [9].

Both functions of NMD, to eliminate aberrant mRNAs and to modulate gene expression have been shown to affect the outcome of several diseases. For example, NMD can prevent the expression of potentially deleterious proteins that might confer a dominant negative phenotype, as was shown for β-thalassemia [14]. Germline mutations in the NMD factor gene *UPF3B* that abrogate normal UPF3B function cause various forms of intellectual disability and other mental disorders [15,16,17,18]. Furthermore, considering the accumulation of cMNR-mutated mRNAs in MSI tumor cells, it is not surprising that NMD plays a significant role in modulation of this cancer phenotype [19,20]. By suppressing the expression of mutated proteins NMD is believed to aid tumor cells to escape the immune system [20]. On the other hand, mutations that disrupt NMD functions are commonly observed in pancreatic cancer [21]. Furthermore, reduction in NMD activity can affect the clinical outcome of hepatocellular carcinomas [22]. It is, therefore, reasonable to assume that cMNR mutations in NMD effector genes might be under positive or negative selection pressure, depending on their effect on NMD efficiency [23]. To test this hypothesis, we aimed to identify coding region microsatellites in NMD effector genes that might be targets of frameshift mutations and explore their impact on the molecular phenotype of MSI tumor cells as defined by their proteomic and phosphoproteomic profile.

## 2. Results

### 2.1. Identification of NMD-Related Genes Harboring cMNRs

We searched our human cMNR database for potential MSI target genes encoding proteins of the NMD pathway. Among 14 NMD-associated genes with coding region microsatellites we excluded eight genes (*CASC3, EIF4A3, MAGOH, PYM, RNPS1, SMG6, UPF1, Y14*) from further analyses because they contained repeats with a maximum length of six mononucleotides that are known to exhibit low mutation frequencies [24]. In contrast, coding microsatellites of increased repeat length (≥7 mononucleotides) were detected in the remaining six NMD-associated genes *SMG1* (T7, A7), *SMG5* (C7), *SMG7* (A9), *UPF2* (A7), *UPF3B* (T7) and *UPF3A* (A7, A9). Although the *UPF3A* gene contained two cMNRs only the A9 repeat was considered for subsequent analyses because it was retained in all *UPF3A* splice variants. Coding repeat mutations are only of functional relevance if affected genes are expressed in the target tissue. Our search for expression data in several databases revealed that these six cMNR-harboring candidate genes are expressed in colon epithelial cells [25,26].

### 2.2. cMNR Frameshift Mutations in NMD-Associated Genes 

We next investigated the cMNR frameshift mutation frequency of these six NMD-associated genes in MSI colorectal cancer cell lines (*n* = 30). PCR fragment length analysis revealed cMNR mutations in *SMG1, SMG7*, and *UPF3A*. Particularly low frequencies of heterozygous mutations occurred in the first A7 repeat of *SMG1* (11%; 2/18) and in the A9 repeat of *SMG7* (15%; 3/19) (Table 1). These mutation frequencies are well within the expected range for repeats of this type and length. In contrast, a high frequency of 1- or 2-bp deletions was detected in the A9 repeat in the *UPF3A* gene (67.7%; 21/31). The majority of these somatic mutations did affect only one allele, whereas several MSI cell lines also showed biallelic mutations in the *UPF3A* coding repeat (19%; 6/31; Supplementary Table S1). However, with the exception of a single normal colon mucosa specimen (1/101) cMNR mutations were detected neither in several control samples including microsatellite stable (MSS) CRC cell lines (0/20) nor in the peripheral blood of healthy donors (0/68). 

These results suggest a positive selection for *UPF3A* mutations at least in cultured cell lines. Therefore, we extended our analysis to primary MSI tumors associated with Lynch Syndrome including colorectal adenomas (*n* = 27) and carcinomas (*n* = 101) as well as cancers of the endometrium (*n* = 13), stomach (*n* = 13) and upper urinary tract (*n* = 11). Although *UPF3A* cMNR frameshift mutations were observed in each MSI tumor entity examined, frequencies varied considerably among different tissues. In particular, a high frequency of cMNR frameshift mutations occurred in MSI colorectal adenomas (55%, 15/27) and carcinomas (61%, 62/101) whereas much lower frequencies were found in MSI stomach (5/13, 38.5%), endometrial (3/13, 23.1%), and urothelial (1/11, 9.1%) tumors (Table 2). The high *UPF3A* mutation frequency in MSI colorectal carcinomas was independently confirmed in an additional set of 78 CRCs (Table 2, validation set). The *UPF3A* mutation status of these tumors did not correlate with clinicopathological features. Overall, *UPF3A* represents the only NMD-related gene genetically altered in more than 50% of MSI colorectal cancer cell lines and MSI primary colorectal tumors. These mutation data in conjunction with our statistical model [27] are highly predictive for positive selection of *UPF3A* mutations. In addition, biallelic mutations in several MSI CRC cell lines and the occurrence of mutations in pre-neoplastic lesions provide strong evidence for a likely contribution of *UPF3A* mutations to MSI tumorigenesis.

### 2.3. UPF3A Protein Expression in MSI Colorectal Cancer Cell Lines

**Figure 1:**
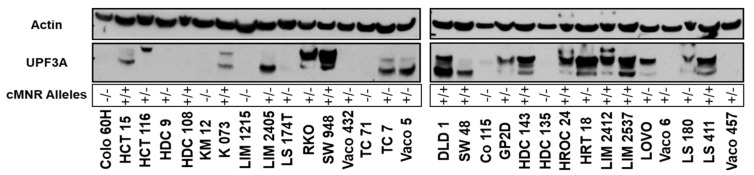
Western Blot analysis of UPF3A protein expression in colorectal cancer cell lines. SW948 cells (MSS) served as UPF3A expression control while ß-actin was used as loading control. The status of wildtype (+) and mutated (−) cMNR alleles for each cell line is indicated.

To determine if the mutational status affects UPF3A expression, we performed Western Blot analyses of protein extracts from 30 MSI colorectal cancer cell lines that differ in their *UPF3A* allele status. As a control we used the colorectal cancer cell line SW948 that has an intact DNA mismatch repair system and lacks the high load of frameshift mutations and truncated proteins. In SW948 cells, protein bands in the expected size range of 56 kDa to 54 kDa were detected by a polyclonal antibody directed against the C-terminus of UPF3A (Figure 1). UPF3A could not be detected in cell lysates of all MSI colorectal cancer cell lines with homozygous A9 cMNR frameshift mutations (*UPF3A*^−^/^−^; 6/30, 20%). Heterogeneous protein expression patterns were observed in MSI cell lines that were either homozygous wild type (*UPF3A*^+^/^+^; 10/30, 33%) or heterozygous mutant for the *UPF3A* A9 coding repeat (*UPF3A*^+^/^−^; 14/30, 47%). Despite the presence of at least one wild type allele, high variation in UPF3A protein expression was observed including complete loss of expression (5/14, 36%) as well as expression of proteins of different sizes detected by the UPF3A antibody (9/14, 64%). We also analyzed UPF3B expression in these cell lines because UPF3A and UPF3B have been proposed to share functional similarity and might regulate each other’s activity [17]. However, no clear correlation between *UPF3A* mutation status and UPF3B expression could be observed (Supplementary Figure S1). 

### 2.4. Expression of UPF3A in Normal Colon Epithelium and Colorectal Tumors

**Figure 2:**
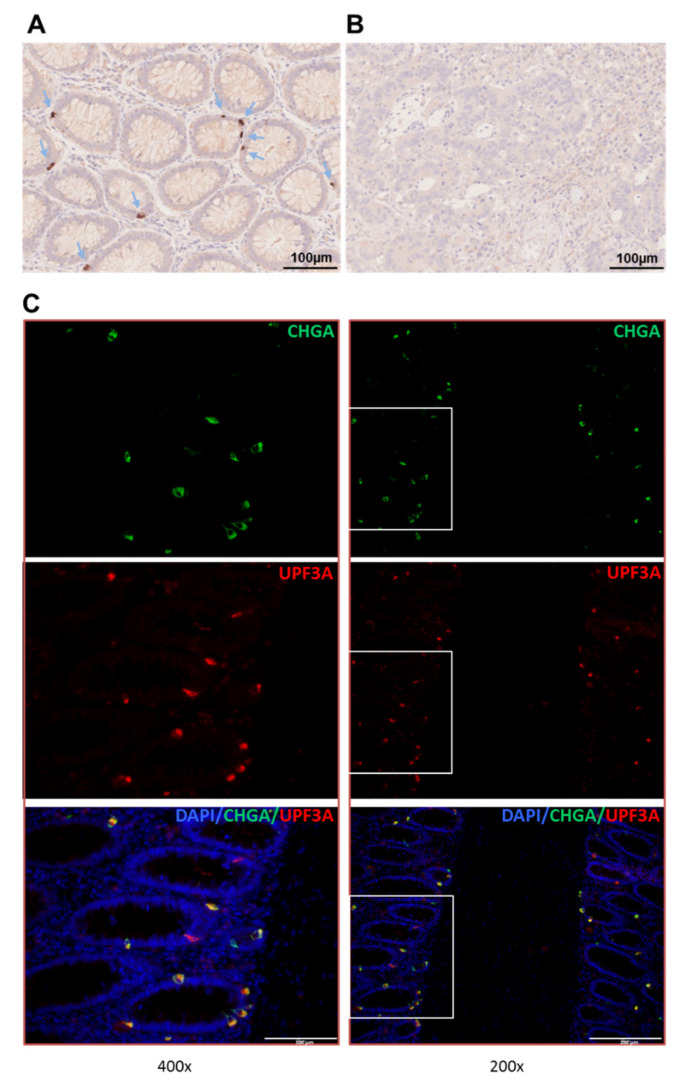
(**A**,**B**) Immunohistochemical staining of UPF3A in normal colon crypts (**A**) and MSI colorectal tumor (*UPF3A* mutated) (**B**). Positively stained cells are indicated by arrows. (**C**) Double immunofluorescence staining of chromogranin A (CHGA; green), a marker of endocrine cells as well as UPF3A (red) in normal colon crypts. Cell nuclei were counterstained with 4′,6-diamidino-2-phenylindole (DAPI). Overlay of the fluorescence signals confirms colocalization of both proteins (yellow and orange) in individual cells.

Information about UPF3A expression in normal colon mucosa is very limited. We therefore performed UPF3A immunohistochemistry on formalin-fixed paraffin-embedded tissue specimens of normal colon epithelium and tumor tissue. Interestingly, strong immunohistochemical staining was found in single cells within the epidermal walls of colon crypts while the surrounding tissue showed very little or no staining (Figure 2A). In contrast, tumor tissues that carry *UPF3A* mutations have lost UPF3A protein expression (Figure 2B). Since the UPF3A staining pattern in normal colonic crypt epithelial cells was reminiscent of the staining pattern known for enteroendocrine cells we performed double immunofluorescence analysis for comparing expression of UPF3A with that of chromogranin A (CHGA), a marker for these terminally differentiated cells (Figure 2C; Supplementary Figure S2). In normal colonic crypts a punctuate pattern of double labeled immunofluorescent cells was observed. In some cells only one of both proteins was expressed. At the subcellular level both proteins showed a slightly different distribution. While UPF3A was expressed basally and thus directed toward the epidermis, CHGA expression overlaps with this site but also extends toward the luminal side of the crypt epithelium. In most labeled cells UPF3A and CHGA expression was predominantly observed in the cytoplasm. These results suggest that UPF3A is expressed in some subset of enteroendocrine cells.

### 2.5. Characterization of KM12-UPF3A Model Cell Line

**Figure 3:**
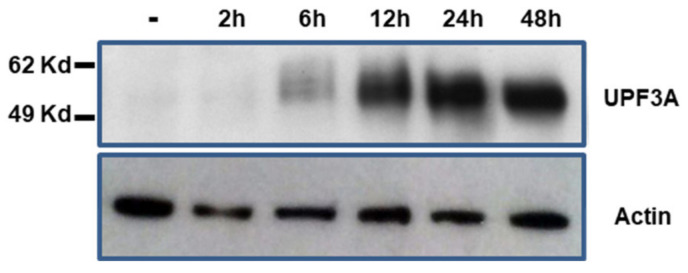
Time course of doxycycline-induced UPF3A protein expression. KM12-UPF3A cells were grown in the absence (−) or presence of doxycline (0.5 µg/mL) for the indicated times and total cellular protein (50 µg) was analyzed by Western blotting. Human β-actin was used as loading control.

Because NMD regulates normal and pathological cellular physiology, perturbation of NMD, for example due to loss of expression of an NMD effector would be expected to alter expression profiles of affected cells. To analyze UPF3A-specific effects, we generated a genetically modified MSI colon cancer cell line KM12-UPF3A (*UPF3A*^−^) that confers doxycycline (dox)-regulated wild type (WT) UPF3A expression in an isogenic background. RT-PCR analysis confirmed expression of the endogenous UPF3A mutant (A8 repeat) as well as the transgenic *UPF3A* WT transcript (A9 repeat) in these cells. At the protein level, KM12-UPF3A cells showed dox-inducible expression of WT UPF3A recognized by the UPF3A antibody on Western blots upon induction and time-course analysis (Figure 3). However, a truncated UPF3A protein predicted to be encoded by the endogenous frameshift mutant *UPF3A* transcript could not be detected, although the antibody used for immunoblotting recognizes an N-terminal UPF3A epitope. Doxycycline itself did not cause any unspecific effects because KM12-Tet-on control cells failed to express any UPF3A protein, both in the absence or presence of doxycycline. Thus, our dox-inducible KM12-UPF3A model system represents a versatile tool to identify MSI-specific molecular and cellular alterations associated with UPF3A expression in an isogenic background.

### 2.6. UPF3A Induces Proteomic and Phosphoproteomic Changes

**Figure 4:**
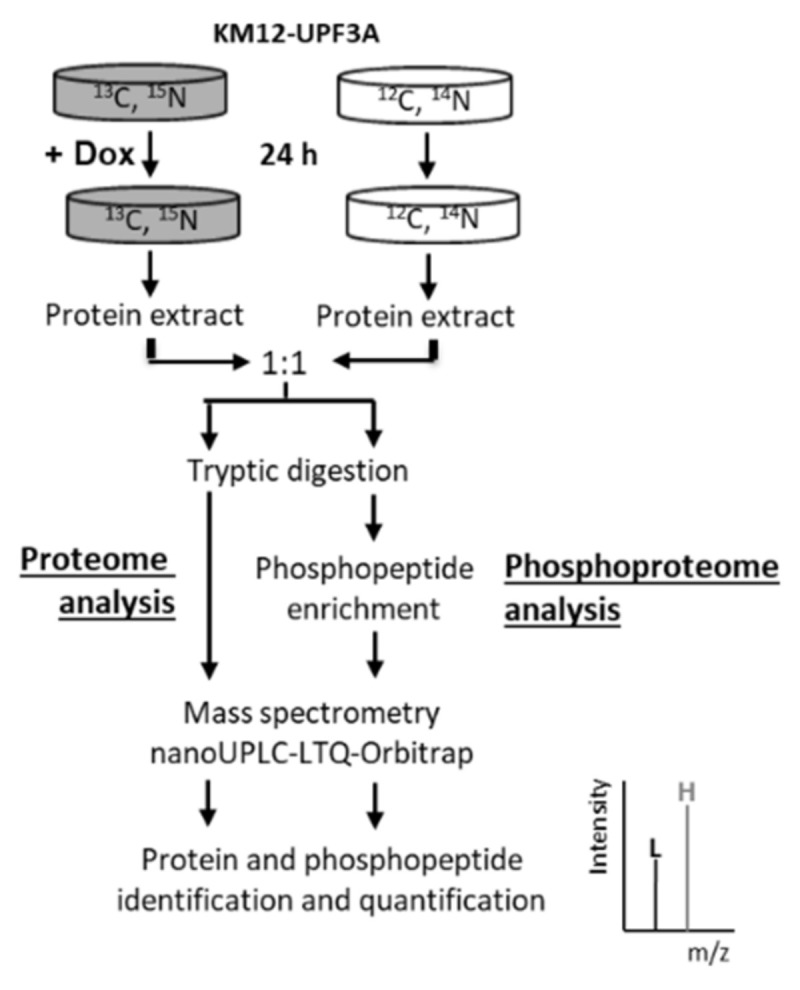
Workflow of proteomic and phosphoproteomic analysis.SILAC labeling with Arg-10 and Lys-8 was applied to KM12-UPF3A cells followed by treatment with doxycycline and mass spectrometric analysis leading to protein and phosphopeptide identification and quantification.

To investigate the consequences of *UPF3A* frameshift mutations on the proteomic landscape of CRC cells, UPF3A-proficient (+dox, pUPF3A), and UPF3A-deficient (dox, dUPF3A), cells were compared by analyzing global protein expression as well as associated phosphorylation changes. Combining proteomic and phosphoproteomic analyses brought the advantage of a deeper understanding of the cell phenotype. Consequently, alterations in protein expression were taken into account in our protein phosphorylation analysis, another advantage of the proposed strategy. SILAC-based proteomic and phosphoproteomic analyses were performed in our KM12-UPF3A model cell line (Figure 4). A total of 1298 proteins were identified and quantified in at least two biological replicates with at least two unique peptides (Supplementary Table S2; Supplementary Figure S3A), including a subset of 35 proteins that were regulated (fold change > 1.5) in a UPF3A-dependent manner (Table 3; Table 4). 

**Figure 5:**
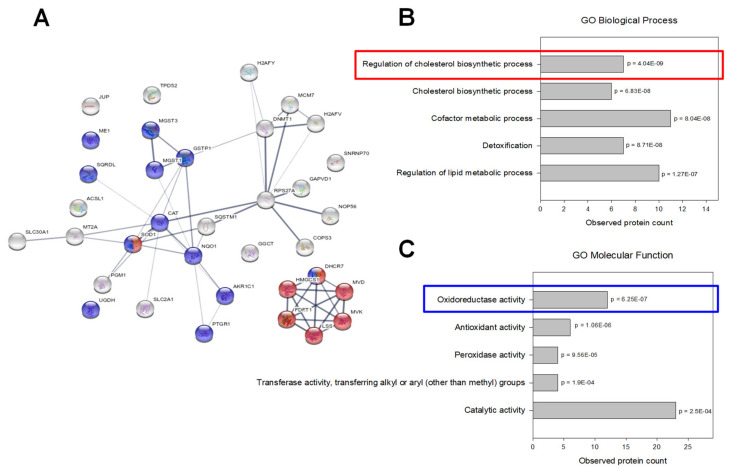
Proteomic data analysis: (**A**) Interaction network of regulated proteins (generated by STRING v11.0). The connecting lines between protein nodes represent protein-protein interactions and the thickness of the edge indicates interaction score (minimum interaction score = 0.4). Coloring of proteins is based on further enrichment analysis. Proteins marked in red are involved in regulation of the cholesterol biosynthetic process. Proteins associated with oxidoreductase activity are marked in blue. (**B**,**C**) Enrichment analysis of regulated proteins (performed in STRING v11.0). Graphs are showing the most enriched Gene Ontology (GO) Biological Processes (**B**) and Molecular Functions (**C**) among the regulated proteins with observed protein count in each category and calculated *p* values corrected for multiple testing (Benjamini and Hochberg).

When these regulated proteins were mapped and visualized by STRING database in an interaction network (Figure 5) and compared with Gene Ontology enrichment analysis, two clusters became apparent: one cluster showed significant enrichment of proteins involved in cholesterol metabolism whereas another cluster centered around proteins with oxidoreductase activity. 

In parallel, 2248 phosphorylation sites were identified with high probability (>0.75) and quantified in at least two biological replicates. To each phosphosite corresponding information from PhosphoSite Plus database was aligned which revealed 27 yet unknown phosphosites (Supplementary Table S3; Supplementary Figure S3B). To avoid false identification of phosphorylation changes due to alterations in whole protein expression, 779 phosphosites have been matched and normalized to their protein expression levels. Among them, 85 phosphosites, located on 52 phosphoproteins, were found to be regulated (>1.5 fold change) in at least two biological replicates (Table 3). The top 10 up- and down-regulated phosphosites are listed in Table 5 and detailed information about each analyzed phosphosite is available in Supplementary Table S4. Interestingly, some phosphosites have been identified on both mono and multiply phosphorylated peptides, sometimes with contrary regulation. One example is the CTNND1 protein. In UPF3A-proficient cells, its S349 site is hypophosphorylated together with two other phosphosites (S346 and S352), but remains hyperphosphorylated when the two adjacent phosphosites have lost their phosphorylation status (Supplementary Figure S4). Similar effects were observed for phosphosites in proteins LMNB1 (S23) and SFSR9 (S213 and S216). 

**Figure 6:**
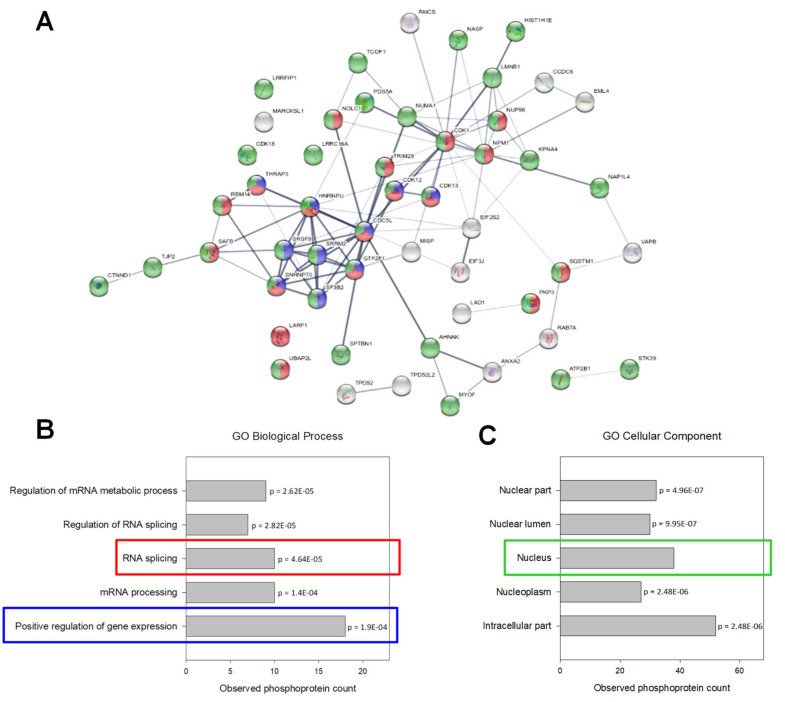
Phosphoproteomic data analysis. (**A**) Interaction network of regulated phosphoproteins (generated by STRING v11.0). The connecting lines between protein nodes represent protein-protein interactions and the thickness of the edge indicates interaction score (minimum interaction score = 0.4). Coloring of proteins is based on further enrichment analysis. Proteins marked in red and blue are involved in RNA splicing and positive regulation of gene expression, respectively. Nuclear phosphoproteins are marked in green. (**B**,**C**) Enrichment analysis of phosphoproteins with regulated phosphorylation (performed in STRING v11.0). Graphs are showing the most enriched Gene Ontology (GO) Biological Processes (**B**) and Cellular Compartment (**C**) among the regulated phosphoproteins with observed protein count in each category and calculated *p* values corrected for multiple testing (Benjamini and Hochberg).

When regulated phosphoproteins were analyzed by Gene Ontology Enrichment and interaction network analysis, a significant proportion of regulated phosphoproteins (75%) comprised nuclear proteins specifically involved in RNA splicing and positive regulation of gene expression (Figure 6).

## 3. Discussion

In microsatellite unstable cells, frameshift mutations in coding microsatellites occur at high frequency often introducing premature translation termination codons into the affected transcripts which thus become potential targets for NMD. As genes encoding NMD factors may contain themselves coding mononucleotide repeats (cMNRs) and as NMD has been shown to be an important modulator of MSI tumorigenesis [19,20], we sought to address the potential link between MSI and NMD by analyzing cMNR mutation rates in NMD factor genes. Focusing only on cMNRs of increased length (>7 nt) we found overall very low mutation frequencies in NMD factor genes with the notable exception of the A9 repeat in *UPF3A*. This indicates that mutations in the overall NMD pathway are not positively selected for, possibly emphasizing the role this pathway plays in preventing an immune reaction against the emerging tumor. This appears to be especially likely as MSI tumors frequently show strong lymphocyte infiltration and a positive selection for mutations in β2-microglobulin thereby facilitating immune evasion [28]. 

UPF3A is one of two human paralogs with homology to the *Saccharomyces cerevisiae* Upf3 protein [29,30] and it has been reported as a potent inhibitor of NMD that can stabilize several substrate mRNAs [10,30,31]. Loss of UPF3A will hence destabilize these transcripts with biological implications if encoded proteins are involved in growth, differentiation, or apoptosis [17]. Therefore, the observed high frequency of *UPF3A* mutations we found in MSI colorectal cancer might actually result from a selective pressure to enhance NMD efficiency. However, preliminary analysis of physiological NMD targets in dUPF3A and pUPF3A MSI CRC cell lines did not reveal such UPF3A-specific effects on NMD efficiency. Although this needs to be corroborated by more detailed experiments it rather suggests that UPF3A may play a role in other processes than NMD.

It is well known that genetic alterations of NMD factors appear to be associated with neuro-developmental disorders. For example, single allele *UFP2* deletions and mutations of *UPF3B* have been identified in patients with intellectual disability (ID) [32]. Similarly, other NMD genes such as *UPF3A*, *SMG6*, *EIF4A3,* and *RNPS1* are frequently deleted and/or duplicated in these patients [33]. It has also been reported that UPF3A is decreased in cells of patients with amyotrophic lateral sclerosis [34]. How altered UPF3A function might contribute to such diverse disease pathologies such as neuronal defects and colon cancer is difficult to reconcile. However, in this context, it is interesting to note that a recent transgenic mouse study uncovered direct physical contact between enteroendocrine cells and neurons innervating the small intestine and colon [35]. Since our data revealed UPF3A expression predominantly in enteroendocrine cells of normal human mucosa, one might speculate that impaired UPF3A function and affected NMD target transcripts disrupt this gut-brain chemosensory circuit leading to abnormal enteroendocrine cell physiology and failure to respond to changes in gut microbiota.

We show for the first time that *UPF3A* frameshift mutations are frequent in MSI colorectal cancer cell lines, primary cancers and adenomas but also occur in MMR-deficient tumors of other organs, albeit at lower frequency. In MSI CRC cell lines, loss of UPF3A expression was associated with biallelic A9 cMNR mutations. However, we did not detect any biallelic *UPF3A* mutations in primary tumor tissues. Despite enrichment of tumor cells by microdissection we cannot exclude that residual inflammatory and connective tissue cells may have confounded mutation analysis. In samples that were found to be heterozygous for frameshift mutations in the A9 coding repeat, additional inactivating point mutations in the remaining *UPF3A* coding sequence also cannot be excluded. Alternatively, haploinsufficiency might impair normal UPF3A function. In fact, even a 50% decrease of Upf3a expression in heterozygous mice (*Upf3a*^+^/^−^) has been reported to be sufficient to cause alterations in NMD substrate levels and defects in spermatocytes [12]. These authors also observed that *Upf3a*^−^/^−^ homozygosity causes embryonic lethality. How complete and/or partial UPF3A loss might contribute to MSI tumorigenesis remains unresolved as long as cell- and tissue-specific normal and aberrant UPF3A expression patterns and their impact on NMD substrates have not been completely elucidated. Our immunohistochemical staining data in normal colon epithelial cells show that UPF3A is not ubiquitously expressed. Instead, overlap of UPF3A expression with staining of the enteroendocrine cell (EEC) marker chromogranin A suggests that in the colon epithelium its expression is restricted to this cell type. Which specific EEC subtype actually is affected by partial or complete loss of UPF3A warrants further investigation.

Apart from these potential target cells of UPF3A expression in the colon epithelium, our proteomics data also uncovered a potential link between UPF3A expression and the cholesterol metabolic pathway. In particular, reconstituted UPF3A expression in our MMR-deficient CRC model cell line was associated with down-regulation of proteins involved in cholesterol biosynthesis and hence UPF3A-deficiency leads to its up-regulation. Cholesterol is an essential building block of cell membranes by modulating membrane fluidity and functions, such as transmembrane signal transduction and interaction with the extracellular matrix. Cellular cholesterogenesis correlates with cell proliferation rates, while suppression of cholesterol biosynthesis inhibits cell growth [36,37,38]. Especially fast growing tumor cells require increased amounts of cholesterol as essential components for membrane buildup, as well as for synthesis of signaling molecules. Accordingly, up-regulation of enzymes for cholesterol biosynthesis causing an increase of cellular cholesterol production is essential for tumorigenesis and tumor progression [39,40,41,42]. Furthermore, analysis of the expression of cholesterol synthesis genes in diverse cancers using the Cancer Genome Atlas (TCGA) also indicated deregulation of cholesterol homeostasis as an important factor in cancer development [43]. Key enzymes of cholesterol biosynthesis are considered to modulate lipid raft structures thereby enhancing raft-associated prometastatic signaling [44]. Several studies specifically elucidated a role of the cholesterol biosynthetic pathway in colon tumorigenesis and progression [45,46,47,48,49] and inhibition of cholesterol synthesis has been suggested for CRC treatment [50,51]. In summary, metabolic reprogramming toward increased cholesterol synthesis appears to be functional in CRC. We for the first time obtained evidence that UPF3A might be involved in this reprogramming process. In this context, loss of UPF3A expression might provide a growth advantage for dMMR CRC tumor cells. However, further relevant experiments should be provided to support this hypothesis.

Our phosphoproteomics data also highlight the impact of UPF3A on the phosphorylation status of nuclear-related proteins. For example, phosphorylation of several Ser residues in the Armadillo family protein CTNND1/p120 were affected by UPF3A expression even in opposing directions. It has been reported that CTNND1 is highly phosphorylated and shuttles between the cytoplasm and nucleus where it can interact with transcriptional activators (β-catenin) and repressors (Kaiso) thereby regulating gene expression [52]. Likewise, CTNND1 is known to be required for nuclear translocation of E-cadherin which in turn regulates β-catenin activity, thereby promoting increased expression of downstream genes and accelerating colorectal tumor growth and migration [53]. To understand how UPF3A activity contributes to the biology of normal and cancerous colon cells more detailed molecular studies are warranted.

Overall, combining computational and mutational screening with inducible gene expression and phospho/proteomic analyses identified UPF3A as a frequent target of frameshift mutations and an important modulator of expression and phosphorylation of proteins involved in cholesterol biosynthesis, redox reactions, and splicing. As a versatile and general approach, it can be applied to any gene and protein of interest.

## 4. Materials and Methods

### 4.1. Database Analyses

Genes involved in NMD were chosen based on the current literature. Candidate genes containing cMNR sequences of at least seven nucleotides in length were identified from seltarbase.org [27]. Existing expression data of candidate genes in colon tissue were obtained from proteinatlas.org [54], biogps.org [25], GeneSapiens system [55] and the genecards.org [26].

### 4.2. Cancer Cell Lines and Human Tissue

Colorectal cancer cell lines were grown under standard conditions in DMEM (Dulbecco’s Modified Eagle Medium) medium (Invitrogen, Carlsbad, CA, USA) supplemented with 10% FCS in the presence of 100 µg/mL penicillin and 100 µg/mL streptomycin (PAA Laboratories GmbH, Cölbe, Germany). Most cell lines and features have been described previously [4,56]. Additional cell lines were provided by: German Cancer Research Center Tumorbank (DLD 1, HCT 15; Heidelberg, Germany), Ludwig Cancer Research Institute (LIM 1215, LIM2 405, LIM 2412, LIM 2537; Melbourne, Australia), M. Schwab (HDC9, HDC 108, HDC 135, HDC143; German Cancer Research Center, Heidelberg, Germany), W. Bodmer (LS411 and GP2D; University of Oxford, UK), S. Michel (K073; Heidelberg University, Germany) and M. Linnebacher (HROC24; University of Rostock, Germany).

Cell growth was determined by the CellTiter 96^®^ AQueous One Solution Cell Proliferation Assay (Promega, Walldorf, Germany) or by cell counting in a Neubauer hemocytometer. Human tissues were obtained from the local tissue bank established within the German Collaborative Group on HNPCC. Two sets of MSI CRCs were analyzed: a test set (*n* = 101) and a validation set (*n* = 78). Clinicopathological features of the validation set are indicated in Supplementary Table S5. Informed consent was obtained from all patients and the study protocol was approved by the local Ethics Committee (Nr. 220/2002, 18 February 2011). For all tissue samples MSI status has been determined previously based on the National Cancer Institute/ICGHNPCC reference marker panel [57] and CAT25 as an additional mononucleotide marker [58]. MSI is defined by instability in at least 30% of tested markers.

### 4.3. Nucleic Acid Isolation, Analysis, and RT-PCR

Genomic DNA was isolated using the DNeasy Tissue Kit (Qiagen, Hilden, Germany). RNA was isolated using the RNeasy Mini Kit (Qiagen). cDNA synthesis was performed with Superscript II Reverse Transcriptase (Life Technologies, Carlsbad, CA, USA) according to the manufacturer’s instructions. For analysis of UPF3A transcripts, fragments of UPF3A cDNA containing the A7 and A9 repeats were PCR-amplified and separated on 2% agarose gels. More detailed analysis of transcript isoforms was performed by cloning different-sized PCR fragments into the pCR2.1 TOPO vector (Life Technologies) and subsequent sequencing. All primers are listed in Supplementary Table S6.

### 4.4. Generation of a Dox-Inducible UPF3A Expression Plasmid

Full-length UPF3A cDNA was PCR-amplified using primers extended by restriction sites NheI and NotI, digested, and cloned into NheI/NotI-digested plasmid pFH-IRESneo [59] resulting in plasmid pFH-IRESneo-UPF3A. From this plasmid the FLAG-HA-UPF3A cDNA sequence was subcloned into KpnI/NotI-digested pTRE-Tight-BI-DsRed-Express vector (Clontech, Mountain View, CA, USA) to yield the final expression vector pTRE-Tight-BI-DsRed-Express-UPF3A.

### 4.5. Stably Transfected Cells

KM12-TET-on colorectal tumor cells were generated by transfecting 10^7^ KM12 cells [60] with plasmid pN1pβactin-rtTA2S-M2-IRES-EG [61] using electroporation (Amaxa Nucleofector 1, program T-20, solution V, 5 μg plasmid DNA). Stable G-418 resistant clones (400 µg/mL) were selected for 4 weeks. Clones were screened for long-term and persistent EGFP (enhanced green fluorescent protein) expression (>9 months) by fluorescence microscopy. EGFP^+^ clones were screened for rtTA activity by transient transfection of a dox-inducible (final dox concentration: 0.5 µg/mL) luciferase reporter gene plasmid [62]. After subcloning by limiting dilution, one KM12-TET-on clone was transfected with plasmid pTRE-Tight-BI-DsRed-Express-UPF3A as described above and stable clones were selected in the presence of hygromycin B (50 µg/mL). Candidate clones were screened for dox-inducible (0.5 µg/mL) expression of DsRed protein by fluorescence microscopy. Finally, one G418- resistant, Hyg-resistant, EGFP-positive, DsRed-positive clone was obtained that showed long-term (>1 year) and persistent dox-inducible UPF3A expression as confirmed by Western blot analysis.

### 4.6. Coding Microsatellite Frameshift Mutation Analysis

Genomic DNA was isolated using the DNeasy Tissue kit (Qiagen). Frameshift mutations were analyzed as described previously [4]. Primers were designed to obtain short amplicons of about 100 bp (Supplementary Table S6) to allow robust amplification from different types of tissue. PCR fragments were analyzed on an ABI3100 Genetic Analyzer (Applied Biosystems, Foster City, CA, USA).

### 4.7. Western Blot Analysis

Soluble protein was extracted by lysing cell pellets in 50 mM Tris-HCl (pH 7.4), 150 mM NaCl, 1% Triton X-100, 1% sodium desoxycholate, 0.1% SDS, 0.1 mM CaCl_2_, and 0.01 mM MgCl_2_, followed by sonication (Bandelin-Sonopuls, Bandelin electronic GmbH & Co. KG, Berlin, Germany) and ultracentrifugation (100,000× *g*, 15 min, 4 °C in a Beckman TLA 100.2 rotor, Beckman Coulter, Indianapolis, IN, USA ). Protein concentration was determined by Lowry assay [63], 65 µg of protein were subjected to Western Blot analysis as described in [64] using the primary antibodies anti-UPF3A (1:5000, rabbit-monospecific, HPA018325, Sigma-Aldrich, Saint Louis, MO, USA) and anti-UPF3B (1:2500, rabbit-monospecific, HPA001800, Sigma-Aldrich), and the secondary antibody anti-Rabbit IgG, HRP Conjugate (Promega). Visualization was performed with Western Lighting Chemiluminescence Reagent Plus (PerkinElmer, Rodgau, Germany) on Kodak BioMax light films (Sigma-Aldrich).

### 4.8. Immunohistochemistry

Paraffin blocks were cut into 2-µm-thick sections. Deparaffinization and tissue staining were performed according to standard protocols [65] using anti-UPF3A primary antibody (1:300 1% horse serum/PBS-T, 2h or overnight at RT; Sigma-Aldrich) followed by secondary biotinylated anti-rabbit antibody (1:50 in 1% horse serum/PBS-T, 30 min at RT; Vector Laboratories, Burlingame, CA, USA).

### 4.9. Immunofluorescence Staining and Imaging

For immunofluorescence staining, 3-µm sections were deparaffinized, rehydrated and boiled in Epitope Retrieval Solution (CINtec^®^ PLUS Cytology, Roche, Basel, Switzerland) for 10 min. After rinsing with deionized water, slides were washed twice in PBS for 5 min and once with deionized water. Immunofluorescence staining was performed as described before [66] with following primary antibodies (in 3% BSA/PBS, overnight, 4 °C): anti-UPF3A (1:300, Sigma-Aldrich) and anti-chromogranin A (1:1000, ScyTek Laboratories, Logan, UT, USA) and secondary antibodies (in 3% BSA/PBS, 30 min at 37 °C followed by 30 min incubation at RT): Alexa Fluor 488 labeled anti-mouse IgG (1:800, Thermo Fisher Scientific, Waltham, MA, USA) and Alexa Fluor 594 labeled anti-rabbit IgG (1:200, Thermo Fisher Scientific). Subsequently, counterstaining of the nuclei with DAPI (1:500, Thermo Fischer Scientific) was carried out in 3% BSA/PBS for 30 min in a humid chamber at RT. Finally, slides were embedded using DAKO Fluorescence Mounting Medium (DAKO, Hamburg, Germany). For colocalization studies both primary as well as secondary antibodies were applied at the same time. Immunofluorescence analysis was carried out using an Olympus AX 70 (40× magnification) microscope. No fluorescence staining was observed on control slides. For further colocalization studies confocal laser scanning microscopy was conducted equipped with a Plan-Apochromat 63×/1.40 Oil DIC objective, a UV diode, Argon and Helium-Neon lasers with emissions at respective wavelengths: 405 nm (DAPI), 488 nm (FITC) and 594 nm (TRITC), as well as reflected light photomultiplier tubes. Image acquisition and processing was performed using Leica LAS AF and ImageJ software [67]. Background was substracted with constant settings using ImageJ’s Rolling ball background subtraction.

### 4.10. SILAC Labeling and Protein Extraction

SILAC labeling was performed as described before [64,68]. Briefly, two cell populations of generated KM12-UPF3A clone were cultured separately in ’heavy medium (containing L-[^13^C_6_, ^15^N_4_] arginine (R10); L-[^13^C_6_, ^15^N_2_] lysine (K8)) or ‘light´ medium (containing L-[^12^C_6_,^14^N_4_] arginine (R0); L-[^12^C_6_,^14^N_2_] lysine (K0)) (Silantes, München, Germany) at 37 °C in a 5% CO_2_ atmosphere for 8 days. To avoid arginine-to-proline conversion, the medium was additionally supplemented with *L*-proline (Sigma-Aldrich) to a final concentration of 200 µg/mL [69]. The saturated incorporation was confirmed as described below and shown at Supplementary Figure S4. ‘Heavy’ labeled cells were then treated for 24 h with doxycycline (500 ng/mL) to induce UPF3A expression, whereas control cell populations were exposed to dox-free medium. The experiment was performed in triplicate. For protein extraction, cells were suspended in a radioimmunoprecipitation assay buffer (RIPA), containing 50 mM Tris-HCl (pH 7.5), 150 mM NaCl, 1% Triton X-100, 0.5% sodium deoxycholate, 0.1% SDS supplemented with 1% DTT and fresh protease (cOmplete Mini; Roche) and phosphatase (PhosSTOP, Roche) inhibitors, and treated with benzonase (125 U; Merck Millipore, Burlington, MA, USA) on an orbital shaker (at 300 rpm) on ice for 1 h. After centrifugation at 13 000 rpm for 30 min at 4 °C protein concentration of the extracts was measured by using 2D Quant Kit reagents (GE Healthcare, Chicago, IL, USA) according to the manufacturer’s instructions.

### 4.11. Tryptic Digestion

Protein lysates from both culture conditions (‘heavy’ and ‘light’) were mixed in a 1:1 ratio based on their protein concentration. Quantitative protein precipitation using a methanol-chloroform-water mixture [70] was performed in order to remove reagents, especially protease inhibitors, prior to tryptic digestion. Enzymatic digestion was performed in low (5 µg) and high (300 µg) protein amount samples as described in [64] with 50 ng or 3 µg trypsin in 40 mM NH_4_HCO_3_ solution overnight at 37 °C (for high protein amount with constant shaking on a Thermomixer (500 rpm)). After digestion, 5 µg protein samples were subjected to shot-gun mass spectrometry analysis, while 300 µg protein samples underwent phosphopeptide enrichment for phosphorylation analysis. In parallel, 5 µg of the protein lysate of ‘heavy’ labeled cells after 8 days of culture was subjected to the analysis of the amino acids incorporation and underwent the same tryptic digestion procedure.

### 4.12. Phosphopeptide Enrichment

IMAC material was prepared as described in [64] from Ni-NTA silica material contained in 6 spin columns (Ni-NTA Spin Columns, Qiagen).

To clean and concentrate peptide mixtures after tryptic digestion, StageTip procedure [71] was applied as described before [64] using C18 material and the reversed phase material (Oligo™ R3, Applied Biosystems) packed into a pipette tip (volume up to 200 µl). Briefly, binding was performed with 2.5% formic acid followed by washing with 2.5% formic acid and elution with 2 times 100 μL of 0.6% acetic acid in 80% acetonitrile.

Each sample was then diluted with 0.6% acetic acid to a final concentration of 60% acetonitrile and added to the 300 µg of prepared IMAC material that was washed thrice with 100 μL of 0.6% acetic acid in 60% acetonitrile before use. Samples were vortexed briefly and incubated for 1.5 h on a rotator. After centrifugation the supernatant from each sample was transferred to the 3 mg of prepared and freshly washed IMAC material (as described above) and incubated on a rotator. After 1.5 h of incubation all IMAC material was washed three times with 100 μL 0.6% acetic acid in 60% acetonitrile. Elution of the phosphopeptides from the IMAC material was performed twice with 40 μL 1% NH_3_ and 5 min incubation with occasional vortexing. The final solution was processed by StageTip purification performed as described above with volume adjustment for lower peptide amount to 10 µL pipette tip (maximal solution volume: 50 µL). Each solution was dried completely in a vacuum centrifuge and frozen. Prior to nanoLC-ESI-MS/MS analysis peptides were redissolved in 5 μL in 2.5% hexafluoroisopropanol/0.1% TFA by sonication for 5 min.

### 4.13. LC-MS/MS

Peptides from tryptic digestion were separated using the Dionex UltiMate 3000 nanoUPLC system as described before [72]. Peptides were trapped on an Acclaim Pepmap 100 column (100 μm × 20 mm, particle size 5 μm). The liquid chromatography separation was performed on a C18 column (75 μm × 50 cm, particle size 2 μm) with a flow rate of 300 nL/min using a 2 h gradient of solvent A (99.9% water, 0.1% formic acid) and solvent B (80% acetonitrile, 19.9% water, 0.1% formic acid) in the following sequence: 2 min at 2% B, from 2 to 8% B in 1 min, from 8 to 25% B in 80 min, from 25 to 40% B in 10 min, from 40 to 95% B in 1 min, 5 min at 95% B, from 95 to 2% B in 1 min, and 20 min at 2% B. The nanoUPLC system was coupled online to a Q Exactive HF-X Hybrid Quadrupole-Orbitrap mass spectrometer (Thermo Fisher Scientific). The following parameters were set: ESI voltage 2200 V; capillary temperature 275 °C, normalized collision energy 35 V. Data were acquired by scan cycles of one FTMS (Fourier-transform mass spectrometry) scan with a resolution of 120,000 at m/z 200 and a range from 300 to 2000 m/z in parallel with ten MS/MS scans in the ion trap of the most abundant precursor ions.

Peptides after phosphopeptide enrichment and peptides for incorporation analysis were analyzed by a linear ion trap quadrupole LTQ Orbitrap-XL mass spectrometer (Thermo Fisher Scientific) coupled to a nanoAcquity ultra high-performance liquid chromatography (UPLC) system (Waters) as described in [64,72]. Peptides were separated on a BEH C18 (100 μm × 100 mm, particle size 1.7 µm), analytical column at a constant flow of 0.4 μL/min using a 3h stepped linear gradient of solvent C (98.9% water, 1% acetonitrile, 0.1% formic acid) and solvent D (99.9% acetonitrile and 0.1% formic acid) in the following sequence from 0 to 4% D in 1 min, from 4 to 25% D in 139 min, from 25 to 40% D in 15 min, from 40 to 85% D in 10 min, 5 min at 85% D, from 85 to 4% D in 2 min, and 15 min at 4% D. The Orbitrap was operated with the following parameters: ESI voltage 2000 V, capillary temperature 200 °C, normalized collision energy 35 V. Data were acquired using XCalibur (version 2.0.7; Thermo Fisher Scientific) by scan cycles of one FTMS scan with a resolution of 60,000 at m/z 400 and a range from 300 to 2000 m/z in parallel with six MS/MS scans in the ion trap of the most abundant precursor ions.

### 4.14. Protein and Phosphopeptide Identification and Quantification

The MS files were processed with the MaxQuant software (version 1.6.2.6) [73] and searched with Andromeda search engine [74] against the human SwissProt database (download: 2019.03.01, 20,412 entries) [75]. Enzyme specificity was set to that of trypsin, allowing for cleavage N-terminal to proline residues and up to two missed cleavage sites (for proteome) and up to four missed cleavage sites (for phosphopeptides). A minimum peptide length of seven amino acids was required. Carbamidomethylation (C) was set as fixed modification, whereas oxidation (M), deamidation (NQ), protein N-terminal acetylation and if necessary, phosphorylation (STY) were considered to be variable modifications. No labeling or double SILAC labeling was defined according to a maximum of 3 or 5 labeled amino acids. Mass tolerances were defined for precursor and fragmented ions as follows: MS first search—20 ppm, MS main search—6 ppm and MS/MS—0.5 Da. The false discovery rates (FDRs) at the protein and peptide level were set to 1%. SILAC-based quantification was based on unique and razor peptides only, and a minimum of two ratio counts was required. Peptide ratios were calculated and normalized for each arginine- and/or lysine-containing peptide as described [73]. In addition, the “match between the runs” feature was implemented with default settings to increase the number of quantified peptides.

Incorporation efficiency of SILAC labeling was analyzed using written R script as described in [76] for all peptides and for peptides with each isotope separately as shown in Supplementary Figure S4.

Further data analysis was performed in Perseus (version 1.6.1.3) software. Matches to the reverse database proteins identified by one site only in modified peptides and common contaminants (KRT2 and KRT10) were removed from the MaxQuant output. Exclusively phosphosites quantified in at least 2 (out of 3) replicates and with localization probability higher than 0.75 were subjected to further analysis. Only proteins identified with at least two unique peptides and quantified in at least 2 (out of 3) biological replicates were considered for the subsequent analysis. Obtained phosphopeptides ratios were corrected for differential protein expression by dividing by the matched protein ratios. Proteins and phosphosites changed by >1.5-fold in at least two biological replicates were considered regulated. In addition, to each identified and quantified phosphosite information from PhosphoSite Plus database [77] were assigned, including known and regulatory phosphosites.

### 4.15. Data Analysis

Global interaction network of regulated proteins and phosphoproteins was predicted in STRING v11.0 [78]. Each protein-protein interaction (PPI) has a combined score (edge score), which represents the reliability of the interaction between proteins. The PPI interactions with a combined score (0: lowest confidence; 1: highest confidence) larger than 0.4 were used for network visualization. In addition, enrichment analysis of regulated proteins and phosphoproteins were performed also in STRING v11 for Gene Ontology Biological Processes (GOBP), Cellular Compartments (GOCC) and Molecular Function (GOMF). Multiple hypothesis testing was controlled by using a Benjamini-Hochberg FDR. Visible clusters on PPI maps were assigned to enriched ontologies by color coding the nodes (proteins and phosphoproteins).

## Figures and Tables

**Table 1:** Frameshift mutation frequencies in NMD-related genes.

Hugo ID	Role in NMD	cMNR	Position ^a^	Mutated
***SMG1***	UPF1 kinase	T7	1339	2/18 (11%)
		T7	9018	0/19
		A7	10694	0/19
***SMG5***	Promotes UPF1 dephosphorylation	C7	12	0/19
***SMG7***	Promotes UPF1 dephosphorylation	A9	2273	3/19 (15%)
***UPF2***	Binds UPF3A/B, recruits UPF1	A7	355	0/16
***UPF3A***	Binds EJC, recruits UPF2	A7	486 ^b^	0/19
		A9	790 ^b^	18/23 (78%)
***UPF3B***	Binds EJC, recruits UPF2	T7	244	0/19

^a^ Positon of the first base in the repeat within the coding sequence, based on the ENSEMBL transcripts ENST00000276201 (SMG1), OTTHUMT00000046308 (SMG5), ENST00000367537 (SMG7), ENST00000262803 (UPF2), ENST00000375299 (UPF3A) and ENST00000276201 (UPF3B); ^b^ UPF3A splice variant represented by ENSEMBL transcript ENST00000351487 lacks the A7 repeat while the A9 repeat starts at coding base pair 691.

**Table 2:** Frameshift mutation frequencies in different tumor entities for the A9-repeat of *UPF3A*.

Tissue	Colorectal			Endometrial	Gastric	Urothelial
Stage	Cancer	Adenomas	Cell Lines	Cancer	Cancer	Cancer
**Mut**	62/101 (61%) ^a^ 51/78 (65%) ^b^	15/27 (55%)	21/31 (67%) ^a^	3/13 (23%)	5/13 (38%) ^a^	1/11 (9%)
**Biall. Mut**	–	–	6/31 (19%)	–	–	–

^a^ Test set; ^b^ Validation set.

**Table 3:** Summary of the proteome and phosphoproteome profiling upon UPF3A expression.

Proteins	Total #
Identified and quantified	1298
Regulated *	35
**Phosphosites**	
Identified and quantified	2248
Unknown ^§^	27
Corrected for protein expression	779
Regulated sites (phosphoproteins)	85 (52)
Known regulation ^§^	7

* >1.5-fold in at least two biological replicates; ^§^ According to PhosphoSitePlus.

**Table 4:** UPF3A-induced protein regulation (>1.5 fold in at least two biological replicates).

Protein Name	Gene Name	Ratio pUPF3A/dUPF3A
GTPase-activating protein and VPS9 domain-containing protein 1	*GAPVD1*	2.19
DNA (cytosine-5)-methyltransferase 1	*DNMT1*	1.88
Tumor protein D52	*TPD52*	1.70
COP9 signalosome complex subunit 3	*COPS3*	1.65
U1 small nuclear ribonucleoprotein 70 kDa	*SNRNP70*	1.64
DNA replication licensing factor MCM7	*MCM7*	1.49
Nucleolar protein 56	*NOP56*	−1.51
Phosphoglucomutase-1	*PGM1*	−1.51
Superoxide dismutase [Cu-Zn]	*SOD1*	−1.53
NAD(P)H dehydrogenase [quinone] 1	*NQO1*	−1.56
Microsomal glutathione S-transferase 1	*MGST1*	−1.56
Core histone macro-H2A.1	*H2AFY*	−1.60
UDP-glucose 6-dehydrogenase	*UGDH*	−1.61
Sulfide:quinone oxidoreductase. mitochondrial	*SQRDL*	−1.67
Glutathione S-transferase P	*GSTP1*	−1.67
Hydroxymethylglutaryl-CoA synthase. cytoplasmic	*HMGCS1*	−1.71
Mevalonate kinase	*MVK*	−1.74
NADP-dependent malic enzyme	*ME1*	−1.75
Solute carrier family 2. facilitated glucose transporter member 1	*SLC2A1*	−1.80
Ubiquitin-40S ribosomal protein S27a	*RPS27A*	−1.80
Catalase	*CAT*	−1.80
Microsomal glutathione S-transferase 3	*MGST3*	−1.80
Diphosphomevalonate decarboxylase	*MVD*	−1.87
Lanosterol synthase	*LSS*	−1.88
Gamma-glutamylcyclotransferase	*GGCT*	−1.90
7-dehydrocholesterol reductase	*DHCR7*	−1.96
Long-chain-fatty-acid--CoA ligase 1	*ACSL1*	−1.97
Histone H2A.V	*H2AFV*	−2.00
Zinc transporter 1	*SLC30A1*	−2.03
Prostaglandin reductase 1	*PTGR1*	−2.11
Squalene synthase	*FDFT1*	−2.52
Aldo-keto reductase family 1 member C1;	*AKR1C1*	−2.90
Sequestosome-1	*SQSTM1*	−3.37
Junction plakoglobin	*JUP*	−4.41
Metallothionein-2	*MT2A*	−5.88

* Mean of ratios from three biological replicates.

**Table 5:** Top 10 up-regulated and top 10 down-regulated phosphopeptides upon UPF3Aexpression.

Protein Name	Gene Name	Phosphosite	Ratio pUPF3A/dUPF3A
Lamin-B1 *	*LMNB1*	T20, S23	2.39
Nucleophosmin	*NPM1*	S254	2.35
Catenin delta-1 ^§^	*CTNND1*	S349	2.15
Cell division cycle 5-like protein	*CDC5L*	S303	2.05
Multifunctional protein ADE2; Phosphoribosylaminoimidazole-succinocarboxamide synthase	*PAICS*	S27	2.01
Neuroblast differentiation-associated protein AHNAK	*AHNAK*	S5763	1.96
General transcription factor IIF subunit 1	*GTF2F1*	S224	1.95
Importin subunit alpha-3	*KPNA4*	S60	1.88
MARCKS-related protein	*MARCKSL1*	S104	1.84
Sister chromatid cohesion protein PDS5 homolog A	*PDS5A*	S1206	1.77
Serine/arginine repetitive matrix protein 2	*SRRM2*	S377	−2.27
Tumor protein D52	*TPD52*	S223	−2.30
Tumor protein D54	*TPD52L2*	S19	−2.71
Serine/arginine repetitive matrix protein 2 *	*SRRM2*	S876	−2.90
RNA-binding protein 14	*RBM14*	S618	−3.20
Serine/arginine-rich splicing factor 9	*SRSF9*	S211, S216	−3.41
Nuclear mitotic apparatus protein 1	*NUMA1*	S1969	−3.43
Catenin delta-1	*CTNND1*	S230	−3.58
Catenin delta-1 ^§^	*CTNND1*	S346, S349, S352	−3.74
Ras-related protein Rab-7a	*RAB7A*	S72	−5.34

* Mean of ratios from three biological replicates; ^§^ Phosphosite (S349) identified on both mono and multiple phosphorylated peptides with different expression levels.

## Supplementary Materials

Supplementary materials can be found at https://www.mdpi.com/1422-0067/21/15/5234/s1.

## Abbreviations

cMNR	Coding mononucleotide repeat
CRC	Colorectal cancer
Dox	Doxycycline
dUPF3A	UPF3A-deficient
FTMS	Fourier-transform mass spectrometry
MMR	DNA mismatch repair
MS	Mass spectrometry
MSI	Microsatellite instability
MSS	Microsatellite stability
NMD	Nonsense-mediated RNA decay
PPI	Protein-protein interaction
PTC	Premature termination codon
pUPF3A	UPF3A-proficient
SILAC	Stable isotope labeling with amino acids in cell culture
UPLC	Ultra high-performance liquid chromatography
WT	Wild type
