# Supplementary material for: (Phospho)proteomic Profiling of Microsatellite Unstable CRC Cells Reveals Alterations in Nuclear Signaling and Cholesterol Metabolism Caused by Frameshift Mutation of NMD Regulator UPF3A
Source: Int J Mol Sci. 2020 Jul 23;21(15):5234. doi: 10.3390/ijms21155234 (PMC7432364; doi:10.3390/ijms21155234)
Supplement: Supplementary file 1 [file ijms-21-05234-s001.zip › SupplFigure_S1.pdf]

Suppl. Figure S1

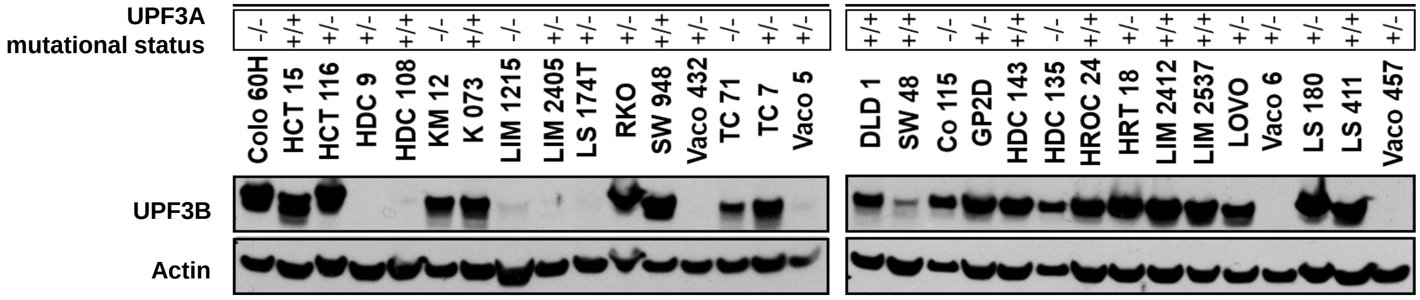

Suppl. Figure S1. Expression of UPF3B in colorectal cancer cell lines. Western Blot analysis of UPF3B protein expression in colorectal cancer cell lines in regard to mutational status of UPF3A alleles. SW948 cells (MSS) served as *UPF3A$^{+/+}$* control while ß-actin was used as loading control.
