# Supplementary material for: (Phospho)proteomic Profiling of Microsatellite Unstable CRC Cells Reveals Alterations in Nuclear Signaling and Cholesterol Metabolism Caused by Frameshift Mutation of NMD Regulator UPF3A
Source: Int J Mol Sci. 2020 Jul 23;21(15):5234. doi: 10.3390/ijms21155234 (PMC7432364; doi:10.3390/ijms21155234)
Supplement: Supplementary file 1 [file ijms-21-05234-s001.zip › SupplFigure_S2.pdf]

Suppl. Figure S2

A

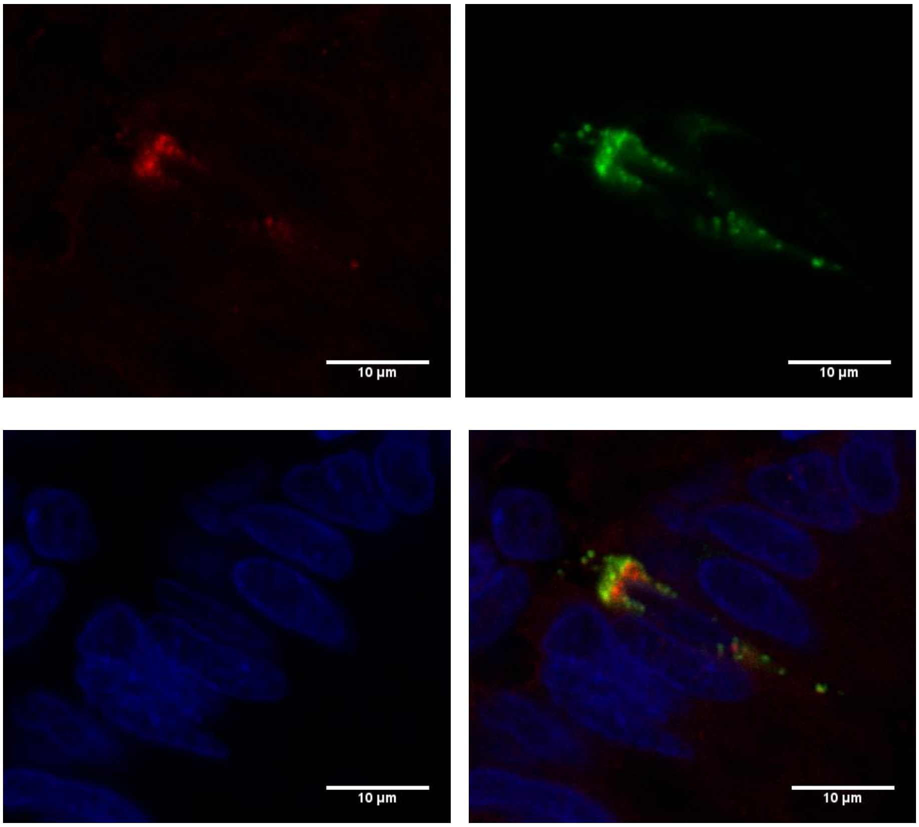

B

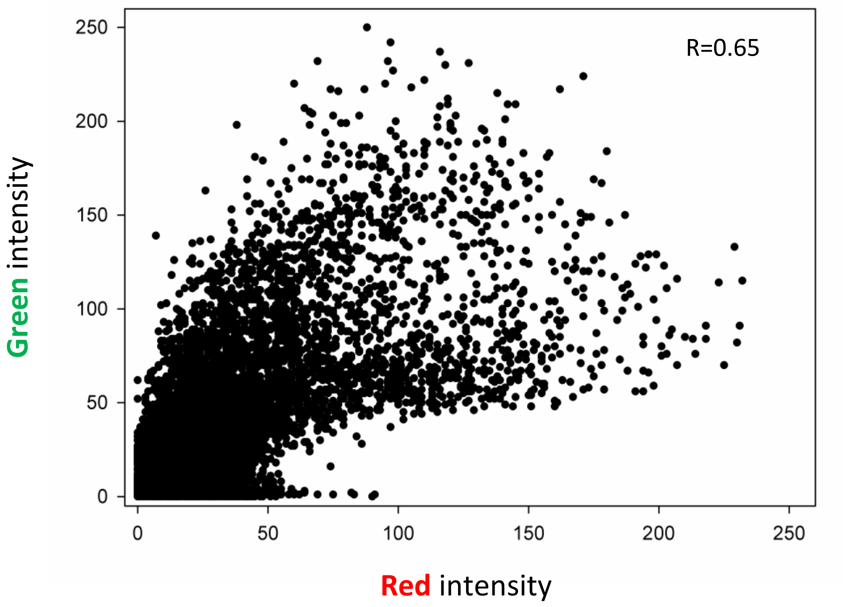

Suppl. Figure S2. Double immunofluorescence staining analyzed by confocal laser scanning microscopy. (A) Localization of UPF3A (Alexa Fluor 598; red fluorescence) and CHGA (Alexa Fluor 488, green gluorescence) in normal colon crypts. Cell nuclei were counterstained with DAPI. Overlay of the fluorescence signals confirms colocalization of both proteins (yellow and orange) in colon crypts. **(B)** Colocalization analysis of UPF3A (red) and CHGA (green).
