# Supplementary material for: (Phospho)proteomic Profiling of Microsatellite Unstable CRC Cells Reveals Alterations in Nuclear Signaling and Cholesterol Metabolism Caused by Frameshift Mutation of NMD Regulator UPF3A
Source: Int J Mol Sci. 2020 Jul 23;21(15):5234. doi: 10.3390/ijms21155234 (PMC7432364; doi:10.3390/ijms21155234)
Supplement: Supplementary file 1 [file ijms-21-05234-s001.zip › SupplFigure_S3.pdf]

Suppl. Figure S3

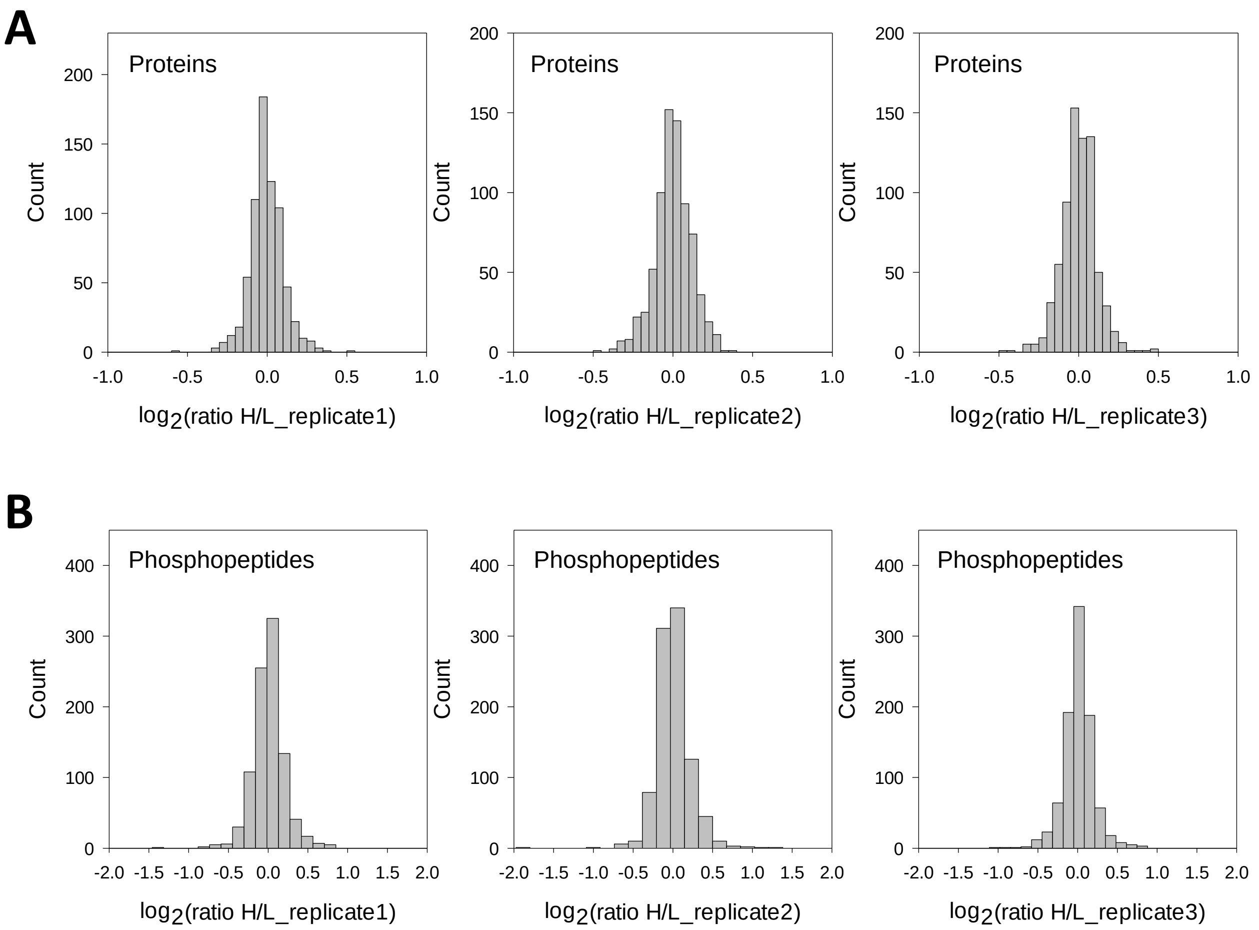

Suppl. Figure S3. Comparison of SILAC-based quantifications between biological replicates. (**A**) 1298 proteins were identified with at least 2 unique peptides and quantified in at least 2 biological replicates. Histogram of each replicate is shown. (**B**) 2248 phosphosites were identified with high confidence and quantified in at least 2 biological replicates. Histogram of each replicate is shown.
