# Supplementary material for: (Phospho)proteomic Profiling of Microsatellite Unstable CRC Cells Reveals Alterations in Nuclear Signaling and Cholesterol Metabolism Caused by Frameshift Mutation of NMD Regulator UPF3A
Source: Int J Mol Sci. 2020 Jul 23;21(15):5234. doi: 10.3390/ijms21155234 (PMC7432364; doi:10.3390/ijms21155234)
Supplement: Supplementary file 1 [file ijms-21-05234-s001.zip › SupplFigure_S4.pdf]

Suppl. Figure S3

A

CTNND1
S349
Ratio pUPF3A/dUPF3A = 2.43*

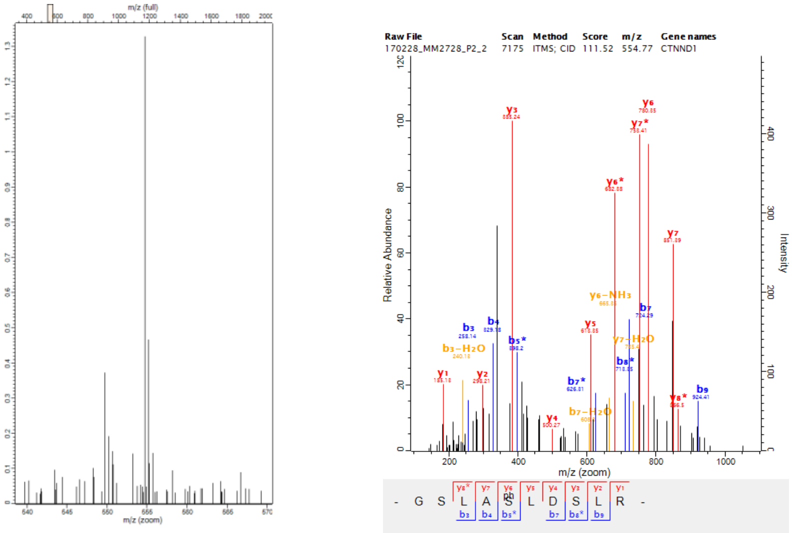

B

CTNND1
S349, S352
Ratio pUPF3A/dUPF3A = -1.41*

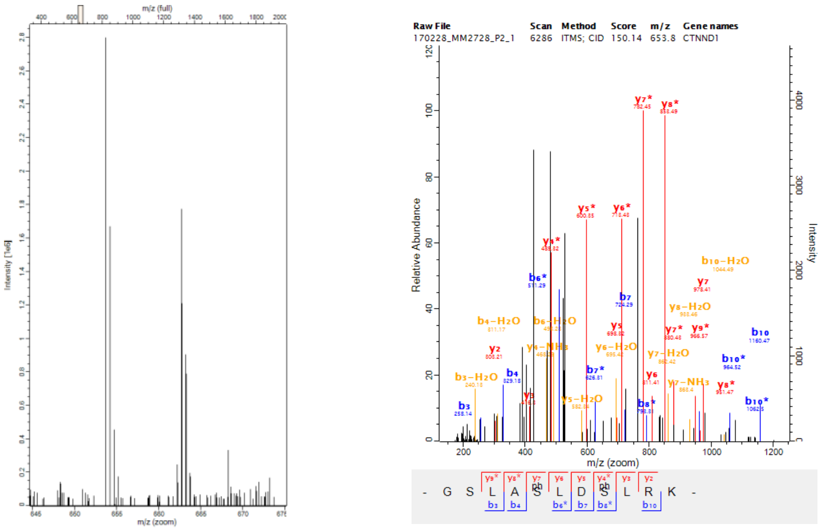

C

CTNND1
S346, S349, S352
Ratio pUPF3A/dUPF3A = -3.45*

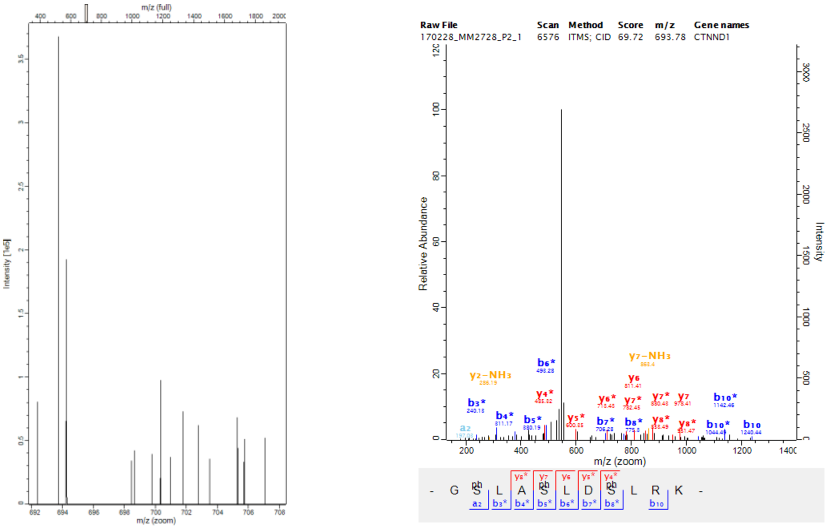

Suppl. Figure S4. Examplary MS1 and MS2 spectra of both mono and multiply CTNND1 phosphosylated peptides with contrary regulation. (A) Mono hyperphosphorylated peptide at S349. **(B)** Doubly phosphorylated peptide at sites S349 and S352. **(C)** Triply hypophosphorylated peptide at sites S346, S349 and S352.

*Mean ratio from three biological replicates. Ratio not corrected for protein expression
