# Supplementary material for: (Phospho)proteomic Profiling of Microsatellite Unstable CRC Cells Reveals Alterations in Nuclear Signaling and Cholesterol Metabolism Caused by Frameshift Mutation of NMD Regulator UPF3A
Source: Int J Mol Sci. 2020 Jul 23;21(15):5234. doi: 10.3390/ijms21155234 (PMC7432364; doi:10.3390/ijms21155234)
Supplement: Supplementary file 1 [file ijms-21-05234-s001.zip › SupplTable_S1.docx]

**Table S1.** Coding Microsatellite (cMNR) Allele Profile of NMD-Associated Genes in MSI-H colorectal cancer cell lines

| **GENE SYMBOL** | ***SMG1*** | | | ***SMG5*** | ***SMG7*** | ***UPF2*** | ***UPF3A*** | | ***UPF3B*** |
| --- | --- | --- | --- | --- | --- | --- | --- | --- | --- |
| **cMNR** | **T7a** | **T7b** | **A7** | **C7** | **A9** | **A7** | **A7** | **A9** | **T7** |
| **CELL LINES** | **ALLELES** | | | | | | | | |
| **Co115** | wt | wt | wt | wt | wt | wt | wt | m1 | wt |
| **Colo60H** | wt | wt | wt | wt | wt | wt | wt | m1p1 | wt |
| **DLD1** | - | - | - | wt | - | - | - | wt | wt |
| **GP2D** | - | - | - | - | - | - | - | m1wt | - |
| **HCT116** | wt | wt | wt | wt | wt | wt | wt | m2wt | wt |
| **HCT15** | - | - | - | - | - | - | - | wt | - |
| **HCT8** | wt | wt | wt | wt | wt | wt | wt | m1wt | wt |
| **HDC108** | wt | wt | wt | wt | wt | wt | wt | wt | wt |
| **HDC135** | - | - | - | - | - | - | - | m1 | - |
| **HDC143** | wt | wt | wt | wt | wt | wt | wt | wt | wt |
| **HDC9** | wt | wt | wt | wt | wt | wt | wt | m2wt | wt |
| **HROC24** | - | - | - | - | - | - | - | wt | - |
| **KM12** | wt | wt | wt | wt | wt | wt | wt | m1 | wt |
| **KO73A** | - | - | - | wt | - | - | - | wt | wt |
| **LIM1215** | - | - | - | - | - | - | - | m1 | - |
| **LIM2405** | - | - | - | - | - | - | - | m1wt | wt |
| **LIM2412** | - | - | - | - | - | - | - | wt | - |
| **LIM2537** | - | - | - | - | - | - | - | wt | - |
| **LIM2551** | - | - | - | - | - | - | - | m1wt |  |
| **LOVO** | wt | wt | wt | wt | wt | wt | wt | m2wt | - |
| **LS174T** | wt | wt | wt | wt | m1wt | wt | wt | m1wt | wt |
| **LS180** | - | wt | wt | - | wt | wt | wt | m1wt | - |
| **LS411** | - | - | - | - | - | - | - | wt | - |
| **RKO** | wt | wt | wt | wt | wt | wt | wt | m2wt | wt |
| **SW48** | m1wt | wt | wt | wt | wt | - | wt | wt | wt |
| **TC7** | m1wt | wt | wt | wt | m1wt | wt | wt | m1wt | wt |
| **TC71** | wt | wt | wt | wt | wt | wt | wt | m1 | wt |
| **VaCo432** | wt | wt | wt | wt | wt | wt | wt | m1wt | wt |
| **VaCo457** | wt | wt | wt | - | m1wt | wt | wt | m1wt | - |
| **VaCo5** | wt | wt | wt | wt | wt | wt | wt | m1wt | wt |
| **VaCo6** | wt | wt | wt | wt | wt | - | wt | m1wt | wt |

wild-type (wt) and mutant alleles; m1: 1bp deletion; m2: 2bp deletion; p1: 1bp insertion
