# Supplementary material for: (Phospho)proteomic Profiling of Microsatellite Unstable CRC Cells Reveals Alterations in Nuclear Signaling and Cholesterol Metabolism Caused by Frameshift Mutation of NMD Regulator UPF3A
Source: Int J Mol Sci. 2020 Jul 23;21(15):5234. doi: 10.3390/ijms21155234 (PMC7432364; doi:10.3390/ijms21155234)
Supplement: Supplementary file 1 [file ijms-21-05234-s001.zip › SupplTable_S6.docx]

**Table S6. Primer Sequences**

| **cMNR Mutation Analysis** | | | |
| --- | --- | --- | --- |
| Gene  Symbol |  | Forward Primer (5’-3’) | Reverse Primer (5’-3’) |
| *SMG1* |  | AGCTGCTTGTTTTCCCCATT | CCGAGCAAAACACCAACAC |
| *SMG1* |  | ACGGTGCTTGGTGGAGTATC | TTGAGCCACCAACTCTTTCTC |
| *SMG1* |  | GGTACCACAGAGCCTGAAGAA | GCTTGGCGAAAGATTGACAT |
| *SMG5* |  | AGTGTTGGCGGTAGTGGTG | GGTAAAGCCGCTTAGTGTGG |
| *SMG7* |  | CAGACCCAAGACCCCATAAA | TGGGACCTTGACTTCTGAGG |
| *UPF2* |  | ACGTTTGGCCTGCTCTTCT | GGAAGACAAGGAACGCAAGA |
| *UPF3A(A7)* |  | TCAGAATGGCTTCTGGAACA | TCGATGCTTCCAGTCTTGG |
| *UPF3A(A9)* |  | GTTTGCGGGAAGAGGAAAA | GGAAATGTTTCCTCAGAATTACCTT |
| *UPF3B* |  | GCATTTCTTAAGGTGGTAATTCG | TTTTTTTTTTACCTCGTATCATTAGA |
|  |  |  |  |
| **RT-PCR Analysis** | | | |
| *UPF3A* |  | TGCCAGCACACGACTACTTC | TCTGTGGTTGGTTCCTCTCC |
|  |  |  |  |
| **cDNA Subcloning** | | | |
| *UPF3A* |  | CCGCTAGCATGCGCTCGGAAAAGGAGGG | GGGGAAGCGGCCGCTCACTCTGC CTCTTCCCTCT |
|  |  |  |  |

Restriction sites (NheI, NotI) in subcloning primers are underlined
